# An unusual elateroid lineage from mid-Cretaceous Burmese amber (Coleoptera: Elateroidea)

**DOI:** 10.1038/s41598-021-01398-w

**Published:** 2021-11-09

**Authors:** Yan-Da Li, Robin Kundrata, Gabriela Packova, Di-Ying Huang, Chen-Yang Cai

**Affiliations:** 1 State Key Laboratory of Palaeobiology and Stratigraphy, Nanjing Institute of Geology and Palaeontology, and Centre for Excellence in Life and Palaeoenvironment, Chinese Academy of Sciences, Nanjing, 210008 People’s Republic of China; 2 Department of Zoology, Faculty of Science, Palacky University, 77146 Olomouc, Czech Republic; 3 School of Earth Sciences, University of Bristol, Life Sciences Building, Tyndall Avenue, Bristol, BS8 1TQ UK

**Keywords:** Palaeontology, Entomology

## Abstract

We here report a new elateroid, *Anoeuma lawrencei* Li, Kundrata and Cai gen. et sp. nov., from mid-Cretaceous Burmese amber. Though superficially similar to some soft-bodied archostematans, *Anoeuma* could be firmly placed in the polyphagan superfamily Elateroidea based on the hind wing venation. Detailed morphological comparisons between extant elateroids and the Cretaceous fossils suggest that the unique character combination does not fit with confidence into any existing soft-bodied elateroid group, although some characters indicate possible relationships between *Anoeuma* and Omalisinae. Our discovery of this new lineage further demonstrates the past diversity and morphological disparity of soft-bodied elateroids.

## Introduction

The beetle superfamily Elateroidea is one of the major and oldest polyphagan groups^1–3^. It contains a broad spectrum of forms; from the lineages with a hard body, clicking mechanism, and five abdominal ventrites of which at least some are connate (e.g., the click-beetles) through variously intermediate groups (e.g., Brachypsectridae and Jurasaidae) to the lineages with an extremely soft body with some morphological reductions, and seven to eight free abdominal ventrites connected with extensive membranes^1,4,5^. Historically, soft-bodied elateroids were thought to form a monophyletic group called cantharoids^6^. Recent molecular studies, however, definitely rejected the monophyly of Cantharoidea^1–3^.

Although the soft-bodiedness is known in several unrelated beetle groups, it is by far most widespread and extensively studied in the superfamily Elateroidea. Soft-bodiedness is hypothesised to be connected with the neoteny, which is probably resulted from the termination or modification of complete metamorphosis^7^. The underlying molecular and developmental mechanisms have not been yet explored for elateroids, but studies in other insects suggested that the changes in a few components in hormone signaling pathway could substantially alter the developmental process and lead to neoteny^8–10^. Although there might be some deep homology in soft-bodied elateroids, different lineages still exhibit diverse morphology and may modify the developmental scheme in various ways^11^. Thus elateroids show a high morphological diversity and developmental plasticity.

Due to the multiple parallelly-evolved morphological traits as a result of incomplete sclerotisation and neoteny in soft-bodied elateroids^1,4,12^, it is almost impossible to properly assess the interrelationships among elateroid families by morphological phylogenetic analyses^11,13^. Even in light of more informative molecular data, the relationships among currently recognised elateroid groups remain unstable^1–3,5,12,14–16^.

Recent years have witnessed the discoveries of several elateroid families of both extant^5,15,17,18^ and extinct forms^11,19^, suggesting a large portion of unknown diversity in Elateroidea. Here, we report a new soft-bodied elateroid from the mid-Cretaceous Burmese amber, which exhibits a unique character combination within the superfamily. We discuss morphology of the newly discovered fossil and compare it to other soft-bodied elateroids.

## Systematic palaeontology

- Order Coleoptera Linnaeus, 1758.
- Suborder Polyphaga Emery, 1886.
- Superfamily Elateroidea Leach, 1815.
- Family *incertae sedis.*

- Genus *Anoeuma* Li, Kundrata & Cai gen. nov.
- urn:lsid:zoobank.org:act:5F05C3D4-2CFA-4DF5-8B0C-CCF4E35BF708.

### Type species

*Anoeuma lawrencei* sp. nov., here designated.

### Etymology

The generic name is an anagram of *Euanoma*, a genus in Elateridae: Omalisinae, in reference of the morphological similarity shared by the new genus and the Omalisinae. Gender: feminine.

### Diagnosis

#### Adult male

Head distinctly hypognathous. Antennae subfiliform, with 11 antennomeres; antennomeres 2 and 3 short. Mandibles unidentate, slender, sickle-shaped. Maxillary palps 4-segmented, with apical palpomere distinctly elongate. Labial palps 3-segmented. Tentorial pits absent. Prosternum in front of coxae subtriangular, longer than diameter of procoxal cavity. Protrochantins large, triangular and with slender process attached to procoxa. Mesocoxae narrowly separated. Elytra short, not completely covering abdomen. Hind wings fully developed, with radial cell closed. Tibial spurs distinct, double. Tarsi simpe, tarsomeres 2–4 relatively short and stout. Abdomen with eight free ventrites.

### Composition and distribution

Monotypic, with *Anoeuma lawrencei* Li, Kundrata & Cai sp. nov. from the Burmese amber (northern Myanmar).

**Figure 1:**
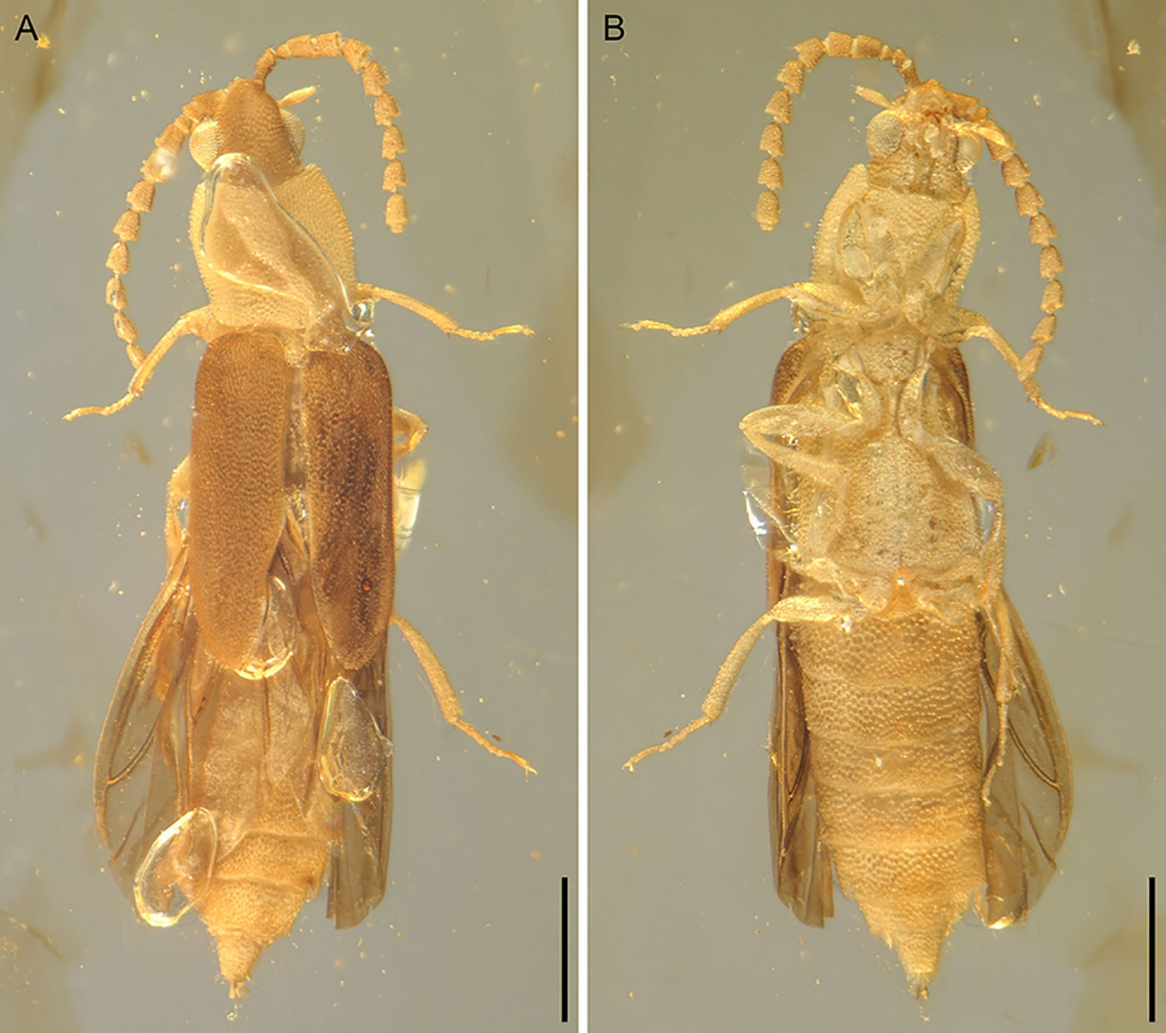
General habitus of *Anoeuma lawrencei* gen. et sp. nov., holotype, NIGP175109, under incident light. (**A**) Dorsal view. (**B**) Ventral view. Scale bars: 500 μm.

**Figure 2:**
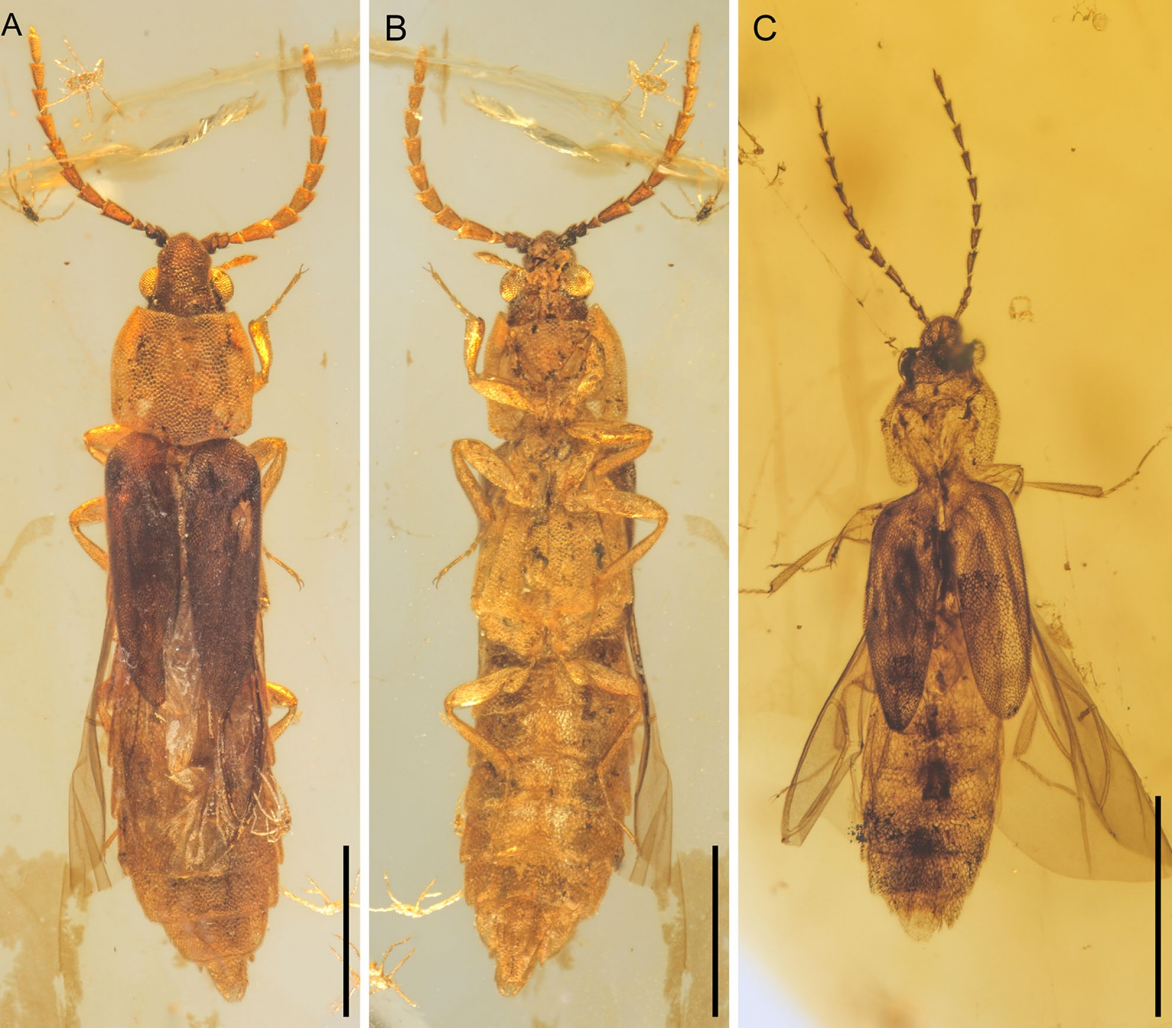
General habitus of *Anoeuma lawrencei* gen. et sp. nov., paratypes, under incident light. (**A**) NIGP175110, dorsal view. (**B**) NIGP175110, ventral view. (**C**) NIGP175111, dorsal view. Scale bars: 1 mm.

**Figure 3:**
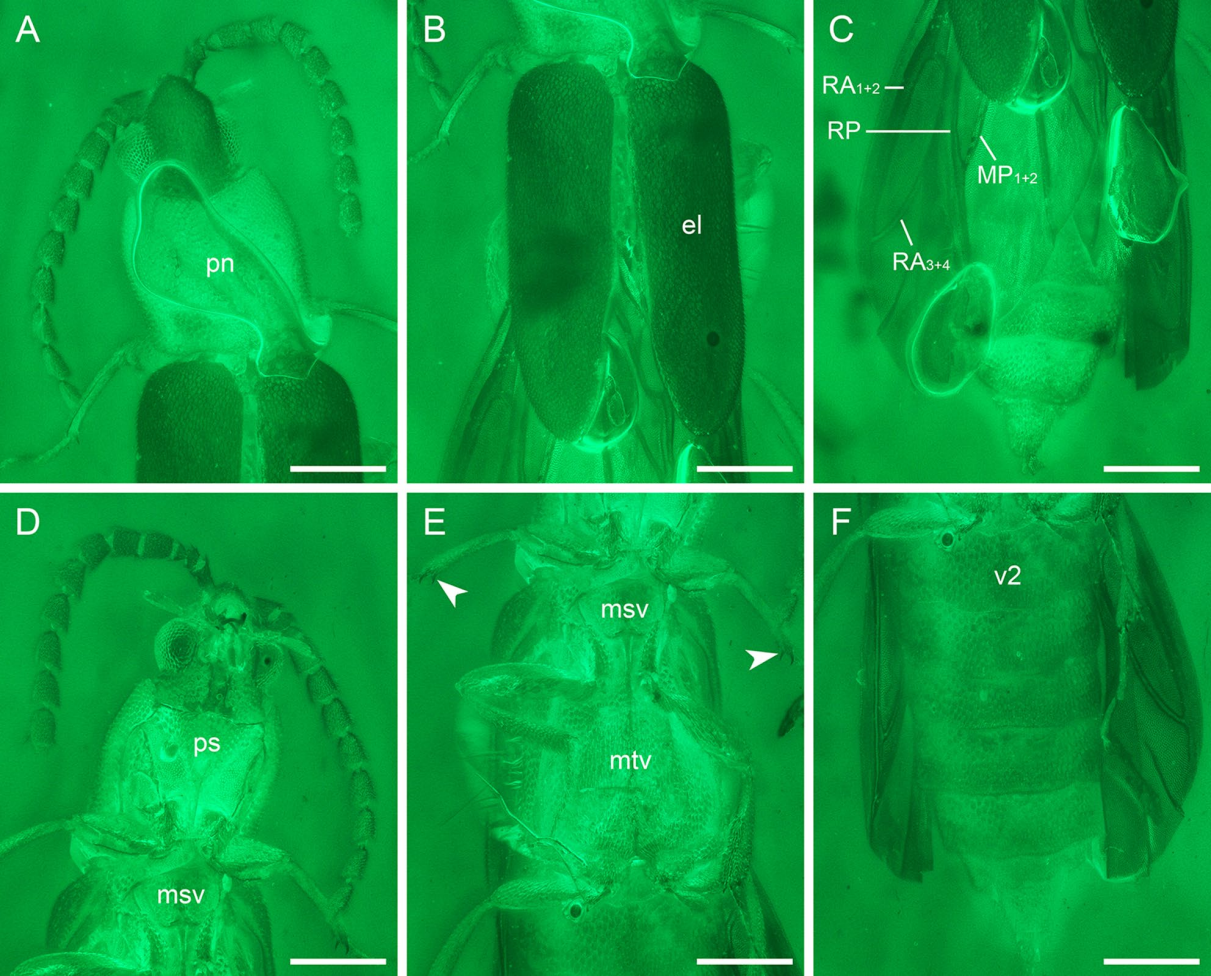
Details of *Anoeuma lawrencei* gen. et sp. nov., holotype, NIGP175109, under widefield fluorescence. (**A**) Head and prothorax, dorsal view. (**B**) Elytra, dorsal view. (**C**) Hind wings and abdomen, dorsal view. (**D**) Head and prothorax, ventral view. (**E**) Meso- and metathorax, ventral view, with arrowheads showing the double tibial spurs. (**F**) Abdomen, ventral view. Abbreviations: el, elytron; msv, mesoventrite; mtv, metaventrite; pn, pronotum; ps, prosternum; v2, ventrite 2. Scale bars: 300 μm.

**Figure 4:**
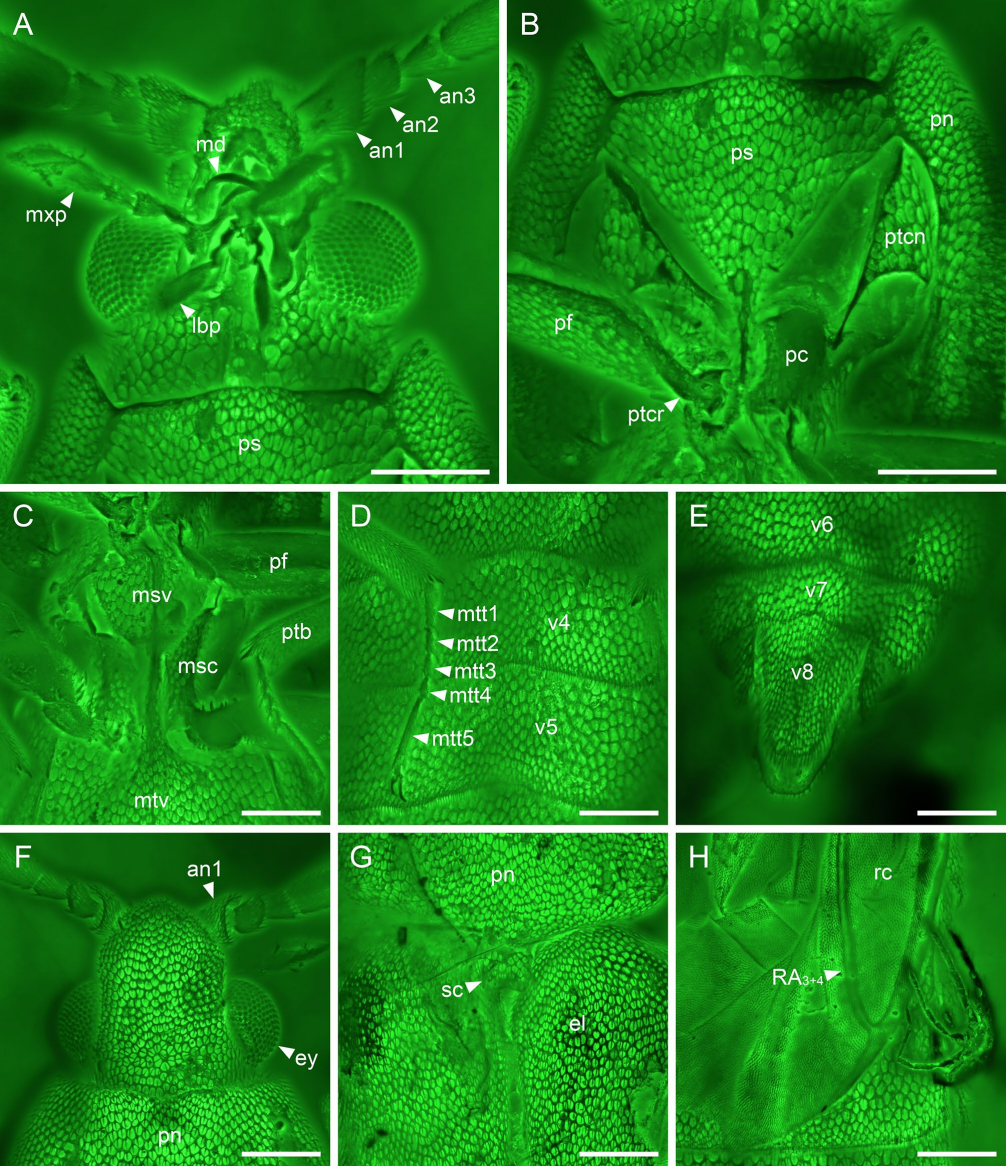
Details of *Anoeuma lawrencei* gen. et sp. nov., paratype, NIGP175110, under confocal microscopy. (**A**) Head, ventral view. (**B**) Prothorax, ventral view. (**C**) Mesothorax, ventral view. (**D**) Middle portion of abdomen, ventral view. (**E**) Abdominal apex, ventral view. (**F**) Head, dorsal view. (**F**) Elytral base, dorsal view. (**F**) Part of hind wing, dorsal view. Abbreviations: an1–3, antennomeres 1–3; el, elytron; ey, compound eye; lbp, labial palp; md, mandible; msc, mesocoxa; mtt1–5, metatarsomeres 1–5; mtv, metaventrite; mxp, maxillary palp; pc, procoxa; pf, profemur; pn, pronotum; ps, prosternum; ptb, protibia; ptcn, protrochantin; ptcr, protrochanter; rc, radial cell; sc, scutellum; v4–8, ventrites 4–8. Scale bars: 200 μm.

**Figure 5:**
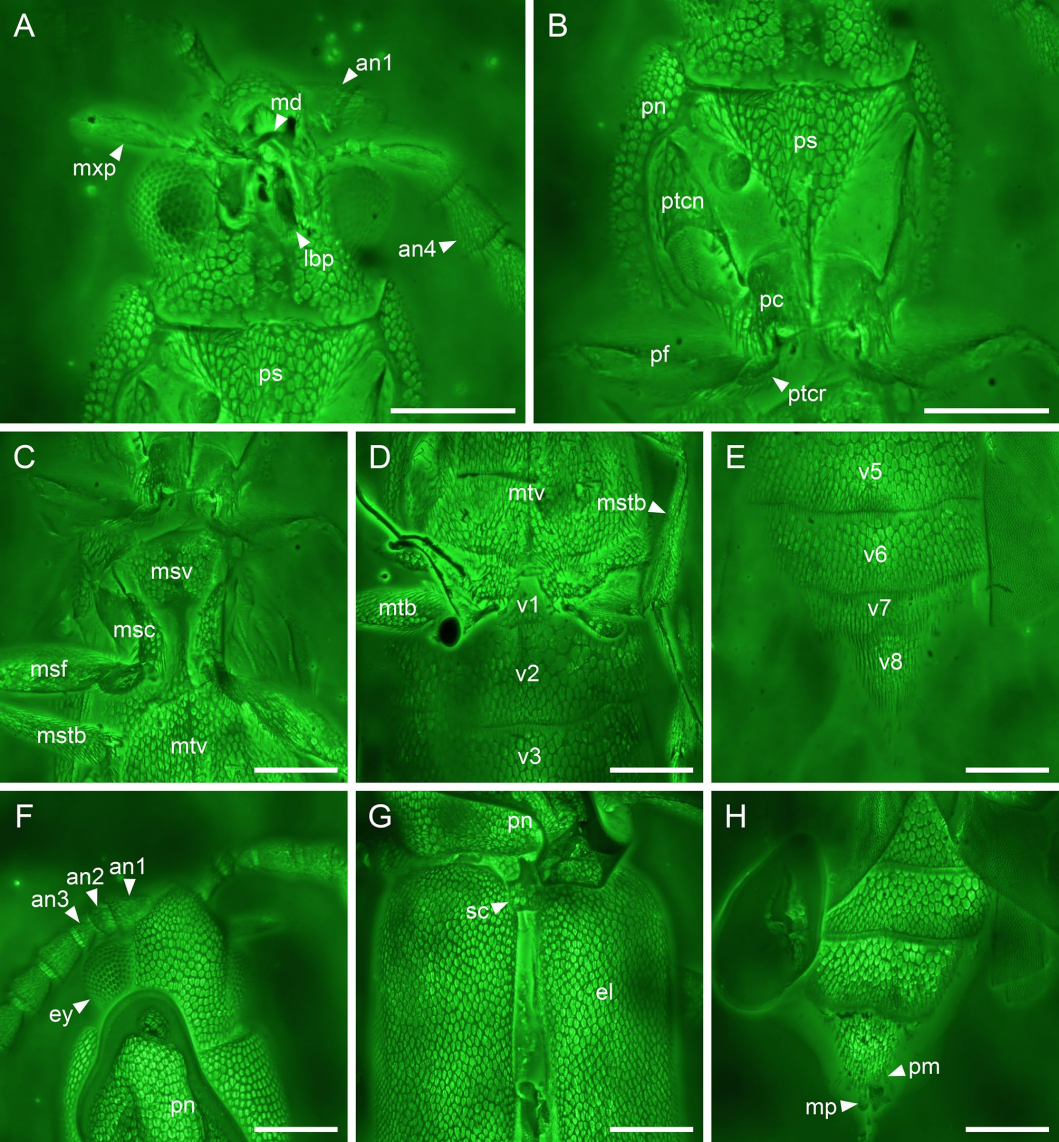
Details of *Anoeuma lawrencei* gen. et sp. nov., holotype, NIGP175109, under confocal microscopy. (**A**) Head, ventral view. (**B**) Prothorax, ventral view. (**C**) Mesothorax, ventral view. (**D**) Abdominal base, ventral view. (**E**) Abdominal apex, ventral view. (**F**) Head, dorsal view. (**F**) Elytral base, dorsal view. (**F**) Abdominal apex, dorsal view. Abbreviations: an1–4, antennomeres 1–4; el, elytron; ey, compound eye; lbp, labial palp; md, mandible; mp, median piece; msc, mesocoxa; msf, mesofemur; mstb, mesotibia; mtb, metatibia; mtv, metaventrite; mxp, maxillary palp; pc, procoxa; pf, profemur; pm, paramere; pn, pronotum; ps, prosternum; ptcn, protrochantin; ptcr, protrochanter; rc, radial cell; sc, scutellum; v1–8, ventrites 1–8. Scale bars: 200 μm.

- *Anoeuma lawrencei* Li, Kundrata & Cai sp. nov.
- urn:lsid:zoobank.org:act:9674500D-16E6-4009-A499-3CE0ECBCDB48.
- (Figs. 1, 2, 3, 4, 5; Supplementary Figs. S1, S2).

### Etymology

The specific name is a patronym in honor of Dr. John F. Lawrence, an internationally recognised coleopterist.

### Type materials

Holotype, NIGP175109, male (NIGP). Five paratypes, males, NIGP175110 (NIGP), NIGP175111 (NIGP), NM-T3471 (NMPC, ex coll. PCRK), BUR003 (PCRK), and BUR004 (PCRK).

### Locality and horizon

Amber mine located near Noije Bum Village, Tanai Township, Myitkyina District, Kachin State, Myanmar; unnamed horizon, mid-Cretaceous, Upper Albian to Lower Cenomanian^20,21^.

### Diagnosis

As for the genus (vide supra).

### Description

#### Adult male

Body weakly sclerotised, elongate, about 4.6–4.9 times as long as wide at humeri, densely punctate and setose.

Head (Figs. 4A,F,5A,F) distinctly hypognathous, almost fully exposed, widest basally, 1.1–1.2 times as long as wide (not including eyes), including eyes almost as wide as anterior margin of pronotum; portion in front of eyes distinctly narrowing anteriad; dorsal surface flat, without protuberance or depression. Compound eyes moderately large and protruding, finely facetted, without interfacetal setae, well separated both dorsally and ventrally. Antennal insertions located fronto-laterally, separated by approximately the maximum width of antennomere 1. Subantennal grooves absent. Antennae with 11 antennomeres, reaching basal third of elytra when directed posteriorly, finely setose; antennomere 1 moderately wide, broadest apically; antennomeres 2 and 3 short and ring-like, subequal in length; antennomere 3 slightly wider than antennomere 2; antennomeres 4–10 moderately elongate, broadest apically; antennomere 11 moderately elongate, fusiform. Ratio of antennomere lengths: ~ 1.8:0.9:1.0:2.2:2.1:2.2:2.2:2.2:2.3:2.2:2.9. Labrum plate-like, widely rounded apically. Mandibles narrow and slender, sickle-shaped, gradually curved mesally with sharply acute apices. Maxillary palps 4-segmented; apical palpomere elongate, about 3.3 times as long as penultimate one. Labial palps 3-segmented; apical palpomere relatively elongate, more than three times as long as penultimate one. Posterior tentorial pits absent.

Pronotum (Fig. 3A) subquadrate, 1.1 times as wide as long; pronotal disc with surface flat, without protuberance or depressions; anterior margin widely concave; anterior angles slightly produced forward; posterior angles rounded, not produced; lateral protonal carinae probably present. Scutellar shield small, transverse, anterior and posterior edges slightly emarginate. Prosternum (Figs. 4B,5B) in front of coxae subtriangular, longer than diameter of procoxal cavity; prosternal process developed, extremely slender, reaching middle of procoxae. Pronotosternal sutures short, slightly curved. Protrochantins (Figs. 4B,5B) large, triangular and with slender process attached to procoxa. Procoxae suboval, slightly emarginate at contact point with protrochantin, contiguous, not strongly projecting. Mesoventrite (Figs. 4C,5C) well-developed, sclerotised; anterior edge not emarginate. Mesocoxae elongate, narrowly separated. Suture between mesoventral process and anterior process of metaventrite invisible. Metaventrite (Fig. 3E) large, with almost complete discrimen; metakatepisternal suture absent. Metacoxae (Fig. 5D) transverse, narrowly separated.

Elytra (Fig. 3B) relatively short, covering only about half of abdomen, together 1.7 times as long as wide, 2.1–2.3 times as long as pronotum; surface irregularly punctate, without carinae; elytral apex rounded. Hind wings (Figs. 2C,3C,4H) fully developed; radial cell closed, not bordered by cross-veins; RP branches not present; RP fused with MP_1+2_ distally.

Legs slender. Trochanters obliquely articulated to femoral bases. Femurs broadest medially. Tibiae each with two distinct spurs. Tarsal formula 5–5-5; all tarsomeres simple, tarsomeres 2–4 relatively short and stout; tarsomere 5 elongate, distinctly longest (Fig. 4D). Claws simple, slightly curved.

Abdomen with eight free ventrites (Figs. 3F,4D,E,5D,E); penultimate ventrite distinctly emarginate medially. Aedeagus trilobate, not fully exposed; median lobe narrow subapically, then gradually widened and rounded apically; parameres with tips simple, narrowly rounded, with long setae (Figs. 1,5H).

### Measurements

NIGP175109 (holotype): BL (body length) 3.19 mm, BW (body width) 0.69 mm, HL (head length) 0.41 mm, HW (head width) 0.35 mm, PL 0.50 mm (pronotal length), PW 0.53 mm (pronotal width), EL 1.16 mm (elytral length). NIGP175110 (paratype): BL 4.52 mm, BW 0.92 mm, HL 0.52 mm, HW 0.48 mm, PL 0.75 mm, PW 0.84 mm, EL 1.59 mm. NIGP175111 (paratype): BL 2.77 mm. NM-T3471 (paratype): BL 3.9 mm. BUR003 (paratype): BL 2.8 mm. BUR004 (paratype): BL 4.4 mm.

## Discussion

### Subordinal and superfamilial placement of *Anoeuma* gen. nov.

The here presented fossil shares a rather typical habitus of soft-bodied elateroids. However, there are some characters which can be confusing if one tries to classify this lineage within the Coleoptera. The most intriguing is the ventral prothoracic structure which might seem to be unusual for Polyphaga, including Elateroidea. In Polyphaga, the propleuron is internallised as a cryptopleuron, while in the other three beetle suborders the propleuron remains external^22^. In *Anoeuma* gen. nov., the sclerite between prosternum and hypomeron (Figs. 4B,5B) could be potentially interpreted as a propleuron. If we follow this scenario, the only reasonable placement for the fossil would be in Archostemata, probably close to an aberrant soft-bodied *Micromalthus* LeConte, 1878. The new fossil shares a series of characters with Micromalthidae, including e.g., the absence of dorsal head protuberances (Figs. 4F,5F), the shortened elytra (Fig. 3B), and the higher number of abdominal ventrites (Fig. 3F)^23^. However, many other characters do not support a position of *Anoeuma* gen. nov. near Micromalthidae at all. For example, Micromalthidae possess mandibles with three vertically arranged teeth, which is an apomorphy of the Micromalthidae + Ommatidae clade^24,25^, while *Anoeuma* gen. nov. has simple mandibles (Figs. 4A,5A). When we look at the hind wing, the folding pattern present in the fossil may look untypical for the polyphagan beetles; however, its hind wing venation looks elateroid-like. The radial cell of Archostemata is bordered by the radial bar, R_3+4_, and two cross-veins, while in Elateroidea (and Polyphaga in general, and also *Anoeuma* gen. nov.) the radial cell is bordered by the radial bar and R_3+4_ only, with no cross-veins (Figs. 2C,3C)^26,27^. Further, in Archostemata, the RP branches are relatively well developed, and RP and MP_1+2_ are connected by two cross-veins, while in Elateroidea (and also in *Anoeuma* gen. nov.), the RP branches are usually completely lost, and RP fuses with MP_1+2_ distally (Figs. 2C,3C). Therefore, we believe the newly discovered fossil should be classified in Elateroidea rather than in Archostemata. The sclerite between prosternum and hypomeron could be interpreted as a well-developed protrochantin (Figs. 4B,5B). Indeed, a similarly shaped protrochantin (i.e., triangular with a slender process attached to procoxa) can be found in some other soft-bodied elateroids (e.g., ^15^).

### Position of *Anoeuma* gen. nov. within Elateroidea

The soft-bodiedness and neoteny originated multiple times within the Elateroidea^1,5,7^. The classification of soft-bodied elateroids based solely on morphology is precluded by the fact that many related groups are differently affected by neoteny, thus being not similar, but on the other hand, some lineages look superficially very similar although they are only distantly related^1^. The morphology-based phylogenetic analyses failed to provide us with the natural classification of the Elateroidea^13^ or to determine the phylogenetic position of a recently described fossil softbodied lineage^11^. Thus, since we cannot reliably use the morphological characters to test the position of *Anoeuma* gen. nov. within the Elateroidea, and the DNA, as an another potential source of data, is not available for the Mesozoic fossil taxa^28,29^, we have to rely on the comparison of the newly discovered fossil with other soft-bodied elateroids.

There are several groups within the soft-bodied elateroids which can be easily ruled out as potential relatives of *Anoeuma* gen. nov. due to their prognathous mouthparts (strongly hypognathous in *Anoeuma* gen. nov.) in combination with various other characters, especially the highly reduced and strongly transverse prosternum (subtriangular and rather long in *Anoeuma* gen. nov.). Such groups include the recently described Jurasaidae^5^, Omethidae (including Telegeusinae)^30–32^, and Phengodidae (including Penicillophorini)^33–35^. Rhagophthalmidae and the recently described fossil Cretophengodidae clearly differ from *Anoeuma* gen. nov. in having antennae with 12 antennomeres^11,36^ (11 antennomeres in *Anoeuma* gen. nov.). Drilini (Elateridae: Agrypninae) have only antennomere 2 short, and have bidentate mandibles and setae on outer basal portions of pretarsal claws^4^ (antennomeres 2 and 3 subequal in length, mandibles unidendate, and pretarsal claws without setae in *Anoeuma* gen. nov.). Cantharidae have usually prognathous head, contiguous mesocoxae, tarsomere 4 expanded and ventrally bilobed^37^ (head hypognathous, mesocoxae separated, and tarsomere 4 simple in *Anoeuma* gen. nov.). Lampyridae have head at least partly covered by pronotum, eyes ususally occupying most of the head, prosternum in front of coxae distinctly transverse, elytra usually not reduced, hind wings absent when elytra reduced, and tibial spurs usually absent or indistinct^38,39^ (head not concealed by pronotum, eyes smaller, prosternum distinctly longer, elytra reduced but with fully-developed hind wings, and tibial spurs present and distinct in *Anoeuma* gen. nov.). Lycidae have mostly prognathous mouthparts but the neotenic groups have hypognathous head with reduced mouthparts similar to *Anoeuma* gen. nov. However, lycids differ from *Anoeuma* gen. nov. in much more reduced, distinctly transverse prosternum, relatively widely separated mesocoxae, and hind wings dramatically reduced when elytra reduced^40,41^.

The here described fossil is most similar in habitus to Penicillophorini (Phengodidae), Telegeusinae (Omethidae), Iberobaeniidae, and Omalisinae (Elateridae). Although the Penicillophorini additionally share with *Anoeuma* gen. nov. the antennae without rami, tiny antennomeres 2 and 3 (most genera), and the reduced elytra with hind wings well-developed^42–45^, and Telegeusinae share with with *Anoeuma* gen. nov. more or less elongate apical maxillary palpomere, short tarsomeres 1–4, and reduced elytra with hind wings well-developed^31,32,46^, both groups differ in the above mentioned characters typical for Phengodidae and Omethidae, respectively. Recently discovered monogeneric Iberobaeniidae share with *Anoeuma* gen. nov. the shape of head, hypognathous mouthparts, and short tarsomeres 1–4. However, Iberobaeniidae can be distingushed by 2-segmented labial palps, pronotum without lateral carinae, not apparently shortened elytra, and much more reduced hind wing venation, with e.g., radial cell vestigial)^15,47^. Representatives of the soft-bodied click-beetle subfamily Omalisinae share with *Anoeuma* gen. nov. the general appearance, the slightly serrate antennae, tiny and ring-like antennomeres 2 and 3, and at least some have also a relatively long prosternum (although differently shaped) and a more developed mesoventrite (e.g., *Cimbrion* Kazantsev^48^). *Anoeuma* gen. nov. is superficially most similar to *Paradrilus* Kiesenwetter in the prolonged frontal part of the cranium with (fronto)laterally inserted antennae, hypognathous mouthparts, and the pronotum without well-defined posterior angles (other omalisine genera have better developed posterior angles)^49^. Nevertheless, the fossil genus differs from Omalisinae in having the apparently narrower frontoclypeal part and even more hypognathous mouthparts, subtriangular prosternum with extremely slender prosternal process (prosternum transverse and without prosternal process or clearly subrectangular in Omalisinae), reduced elytra (all known Omalisinae have elytra long, covering whole or most of abdomen), tarsomeres 2–4 short and distinctly shorter than last tarsomere (Omalisinae usually have elongate tasomeres, with only tarsomere 4 relatively shorter, and last tarsomere never distinctly longer), and the elongate terminal maxillary palpomere, which is much longer than preceding ones (terminal palpomere usually only slightly longer than preceding one and never so long in Omalisinae). Therefore, although *Anoeuma* gen. nov. is in some characters more similar to Omalisinae than to other elateroid groups, and some of the characters which distinguish them are highly plastic in some soft-bodied elateroid lineages (e.g., ^50^), we prefer to place the newly discovered fossil in Elateroidea *incertae sedis*. Discoveries of further taxa related to *Anoeuma* gen. nov. in future would probably help us to better understand the systematic placement of this taxon, which we hypothesise may either represent a separate evolutionary lineage on a family rank or is related to the soft-bodied omalisine click-beetles.

## Conclusions

*Anoeuma* gen. nov. exhibits a mixture of characters known in several neotenic elateroid lineages, but on the other hand, it differs from them in other taxonomically important characters. Additionally, the morphology of ventral prothorax and the dense body punctation are somewhat unique among soft-bodied Elateroidea. In the current state of knowledge, we are unable to place *Anoeuma* gen. nov. into any existing family in full confidence. However, since we refrain here from erection of a new suprageneric rank for *Anoeuma* gen. nov., we tentatively classify it as Elateroidea *incertae sedis*. Neotenics are rarely found not only in the fossil record but also recently in the field, mostly due to their cryptic lives. The flight-capable males usually fly only reluctantly and spend most of the day hidden in the soil or other substrates, and larviform females do not move much at all^5,47,51^. Therefore, every report of a new neotenic lineage is extremely important for understanding the past diversity and morphological variability of soft-bodied elateroids. Since the males of *Anoeuma* gen. nov. exhibit a number of characters which are typical for the lineages where the females are strongly affected by the paedomorphic development^5,41^, we expect that the females, when/if discovered, will be flightless and larviform.

## Materials and methods

### Materials

The Burmese amber specimens studied here were derived from amber mines near Noije Bum (26°20' N, 96°36' E), Hukawng Valley, Kachin State, northern Myanmar. The specimens are deposited in the Nanjing Institute of Geology and Palaeontology, Chinese Academy of Sciences, Nanjing, China (NIGP), the Department of Palaeontology of the National Museum, Prague, Czech Republic (NMPC), and the collection of R. Kundrata, Olomouc, Czech Republic (PCRK). The amber pieces were trimmed with a small table saw, ground with emery papers of different grit sizes, and finally polished with polishing powder.

### Fossil imaging

Photographs under incident light were mainly taken with a Zeiss Discovery V20 stereo microscope. Widefield fluorescence images were captured with a Zeiss Axio Imager 2 light microscope combined with a fluorescence imaging system. Confocal images were obtained with a Zeiss LSM710 confocal laser scanning microscope, using the 488 nm Argon laser excitation line^52^. Images under incident light and widefield fluorescence were stacked in Helicon Focus 7.0.2 or Zerene Stacker 1.04. Confocal images were stacked in Helicon Focus 7.0.2. Images were further processed in Adobe Photoshop CC to enhance contrast.

### Nomenclatural acts

This published work and the nomenclatural acts it contains have been registered in ZooBank. The LSID for this publication is urn:lsid:zoobank.org:pub:B36118CC-136B-4FCD-B320-B4EB04D0EFB9.

## Supplementary Information

Supplementary Information.

## Data Availability

The original confocal data are available in Zenodo repository (https://doi.org/10.5281/zenodo.5553492).
