# Supplementary material for: An unusual elateroid lineage from mid-Cretaceous Burmese amber (Coleoptera: Elateroidea)
Source: Sci Rep. 2021 Nov 9;11:21985. doi: 10.1038/s41598-021-01398-w (PMC8578672; doi:10.1038/s41598-021-01398-w)
Supplement: Supplementary file 1 — Supplementary Information. [file 41598_2021_1398_MOESM1_ESM.pdf]

Supplementary Information for

An unusual elateroid lineage from mid-Cretaceous Burmese amber (Coleoptera: Elateroidea)

Yan-Da Li, Robin Kundrata, Gabriela Packova, Di-Ying Huang, Chen-Yang Cai

List of all Supplementary Information:

Supplementary Figure S1. *Anoeuma lawrencei* gen. et sp. nov., paratype, BUR004, under incident light. (A) General habitus, dorsal view. (B) General habitus, ventral view. (C) Head and prothorax, ventral view.

Supplementary Figure S2. *Anoeuma lawrencei* gen. et sp. nov., paratypes, under incident light. (A) NM-T3471, ventral view. (B) BUR003, dorsal view.

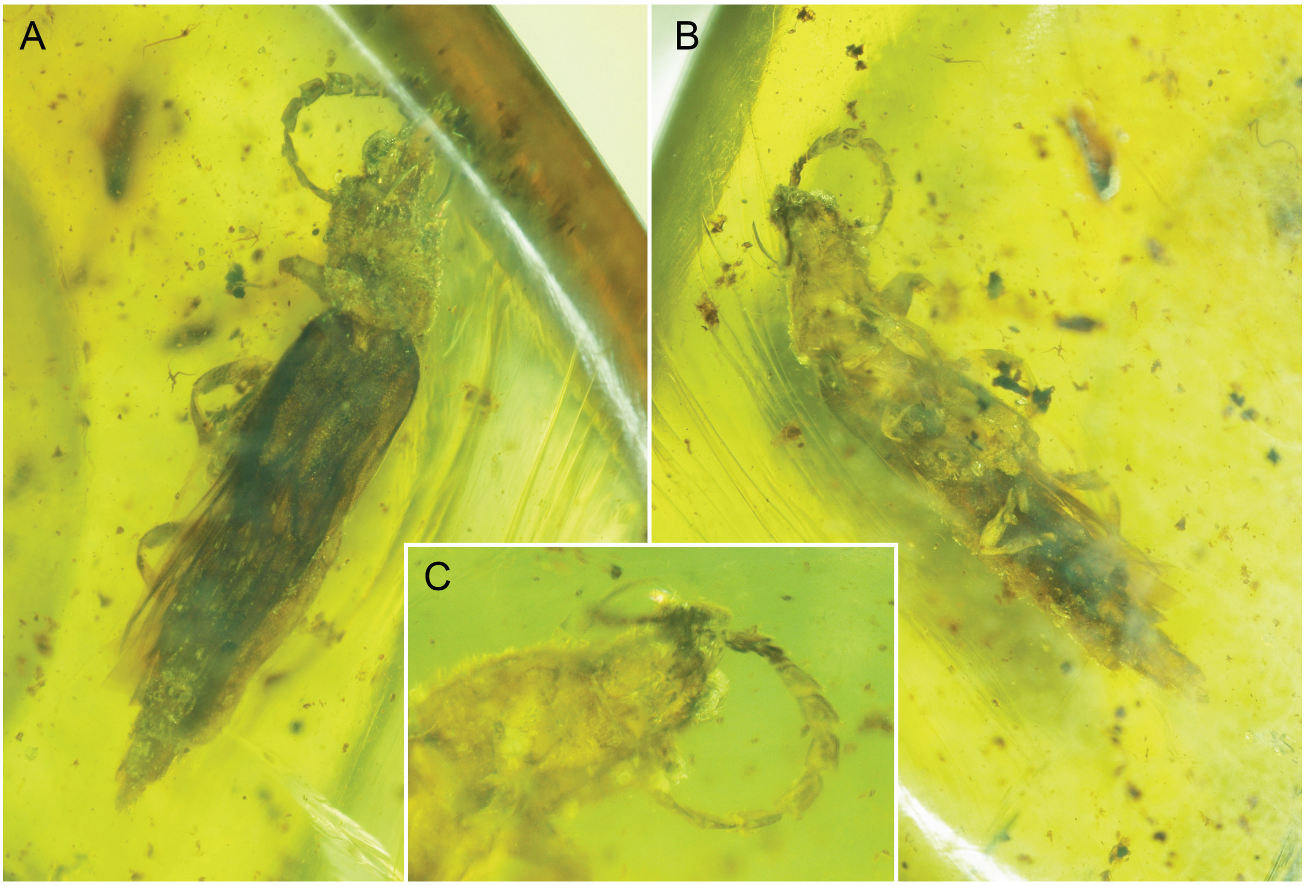

Supplementary Figure S1. *Anoeuma lawrencei* gen. et sp. nov., paratype, BUR004, under incident light. (A) General habitus, dorsal view. (B) General habitus, ventral view. (C) Head and prothorax, ventral view.

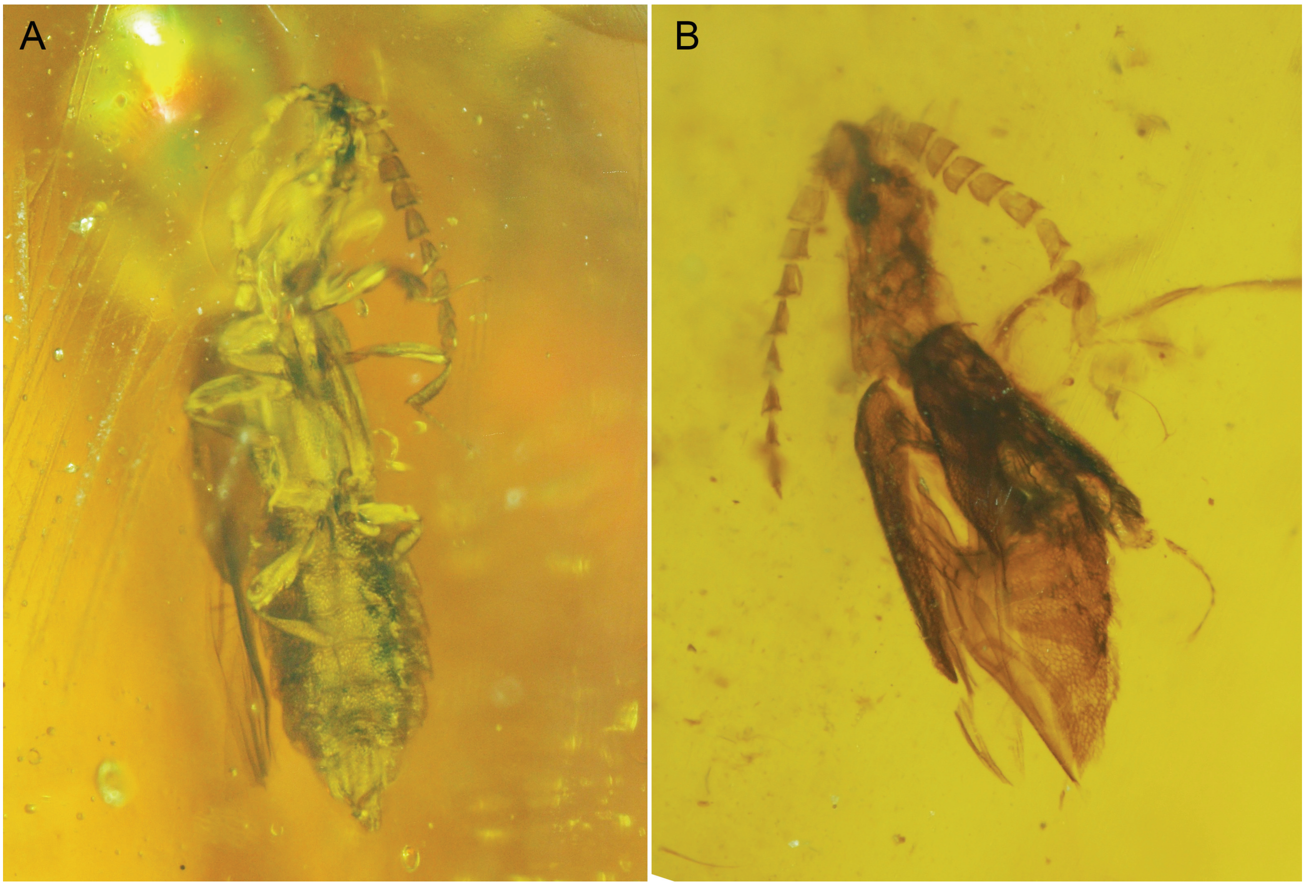

Supplementary Figure S2. *Anoeuma lawrencei* gen. et sp. nov., paratypes, under incident light. (A) NM-T3471, ventral view. (B) BUR003, dorsal view.
